# Improving access to medicines via the Health Impact Fund in India: a stakeholder analysis

**DOI:** 10.1080/16549716.2018.1434935

**Published:** 2018-03-02

**Authors:** Patrick McMullan, Vamadevan S. Ajay, Ravi Srinivas, Sandeep Bhalla, Dorairaj Prabhakaran, Amitava Banerjee

**Affiliations:** ^a^ University of Birmingham Medical School, Birmingham, UK; ^b^ Health System Unit, Centre for Chronic Disease Control, New Delhi, India; ^c^ Research and Information Systems for Developing Countries (RIS), New Delhi, India; ^d^ Training Division, Public Health Foundation of India, New Delhi, India; ^e^ University of Birmingham Centre for Cardiovascular Sciences, Birmingham, UK; ^f^ Farr Institute of Health Informatics Research, University College London, London, UK; ^g^ School of Health, University of Central Lancashire, Preston, UK

**Keywords:** Qualitative, stakeholder analysis, access to medicines, policy, India

## Abstract

**Background**: In India, 50–65% of the population face difficulties in accessing medicines. The Health Impact Fund (HIF) is a novel proposal whereby pharmaceutical companies would be paid based on the measured global health impact of their drugs. We conducted a key stakeholder analysis to explore access to medicines in India, acceptability of the HIF and potential barriers and facilitators at policy level.

**Objectives**: To conduct a stakeholder analysis of the HIF in India: to determine key stakeholder views regarding access to medicines in India; to evaluate acceptability of the HIF; and to assess potential barriers and facilitators to the HIF as a policy.

**Methods**: In New Delhi, we conducted semi-structured interviews. There was purposive recruitment of participants with snowball sampling. Transcribed data were analysed using stakeholder analysis frameworks and directed content analysis.

**Results**: Participation rate was 29% (14/49). 14 semi-structured interviews were conducted among stakeholders in New Delhi. All participants highlighted access to medicines as a problem in India. There were mixed views about the HIF in terms of relevance and scaleability. Stakeholders felt it should focus on diseases with limited or no market and potentially incorporate direct investment in research.

**Conclusions**: First, access to medicines is perceived to be a major problem in India by all stakeholders, but affordability is just one factor. Second, stakeholders despite considerable support for the idea of the HIF, there are major concerns about scaleability, generalisability and impact on access to medicines. Third, the HIF and other novel drug-related health policies can afford to be more radical, e.g. working outside the existing intellectual property rights regime, targeting generic as well as branded drugs, or extending to research and development. Further innovations in access to medicines must involve country-specific key stakeholders in order to increase the likelihood of their success.

## Background

Inadequate access to medicines accentuates worldwide inequalities in health and income [1,2]. Access is dependent on availability, affordability, quality and proper use, and involves a complex interplay between governments, pharmaceutical companies, individuals and society [3–5]. Access to medicines is a problem across many countries, for both communicable and non-communicable diseases, and at several levels [6–8]. India is the second most populous country in the world with 17% of the world’s population but it shoulders 21% of the global disease burden, a major proportion of which requires medicines [9]. However, in 2004, the World Health Organization (WHO) estimated that 50–65% of Indians (499–649 million people) did not have access to even essential medicines [10]. Not only is overall access to medicines limited, but the so-called ‘70–70 problem’ exists, i.e. 70% of Indians’ overall medical expenses are paid upfront and out-of-pocket and 70% of those expenses are on drugs alone [11]. Out-of-pocket drug expenditure pushed 34 million people below the poverty line in 2011 [12].

**Figure 1.**
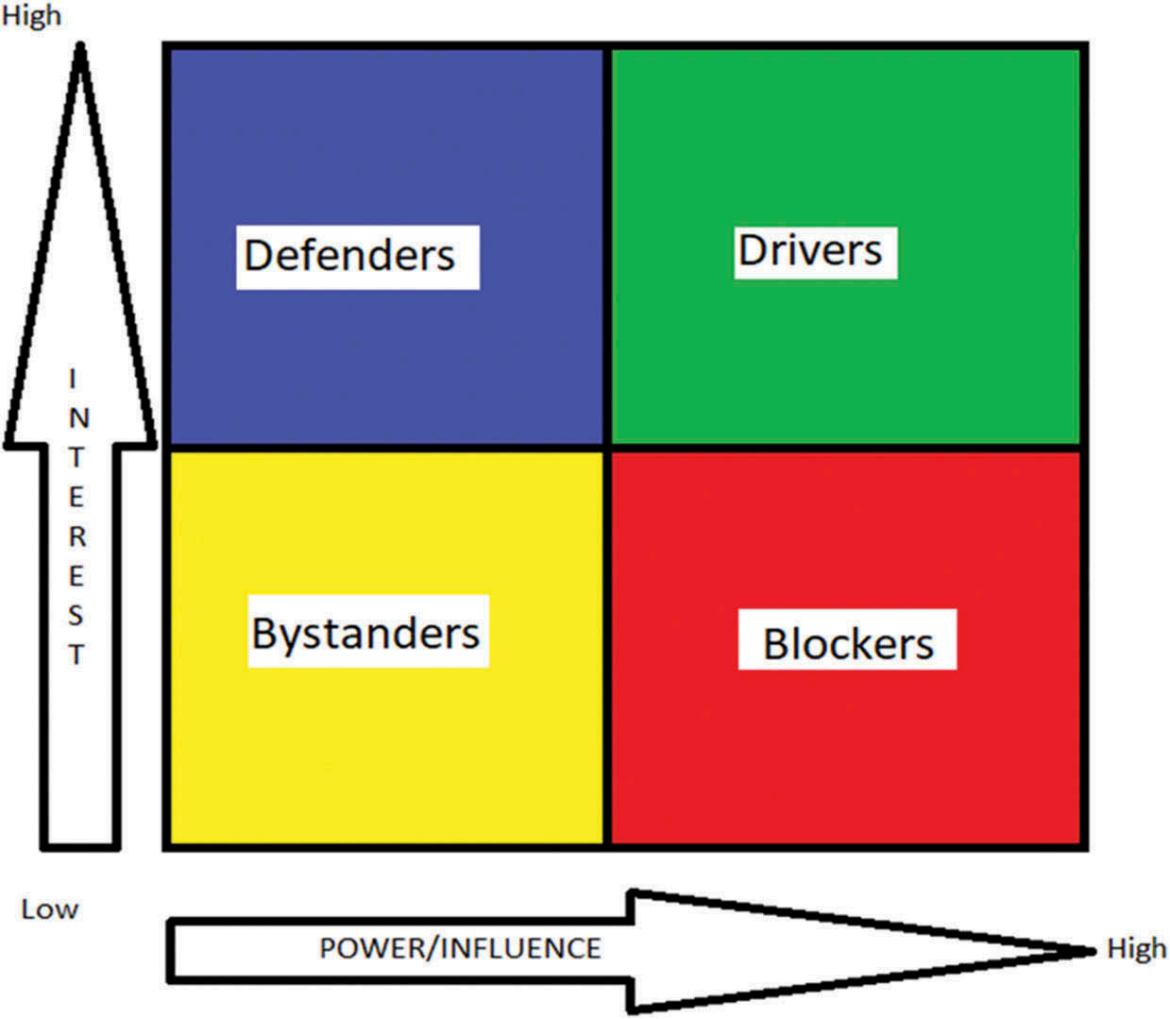
Stakeholder analysis matrix comparing the interest of each actor with their perceived power and influence on health policy.

India has a pharmaceutical industry built upon reverse engineering (analysing products and deducing and developing ways of manufacturing the product from that analysis) and generic production (manufacture of an identical form of the drug to the brand name version) [13]. Since the Trade-Related Aspects of Intellectual Property Rights (TRIPS) agreement was signed by India in 1994 and fully ratified by 2005, the adoption of product-patent law (where intellectual property rests with the product rather than the process of its production) was required and this reverse engineering/generic production model was discarded. The assumption was that the product-patent model would incentivise greater innovation in the pharmaceutical industry and bring about novel medicines at a competitive price [4,13–15]. However, research suggests the contrary [13], and TRIPS may instead reduce access to medicines [16], widening global health inequalities [17,18].

Procurement of essential medicines needs to be improved to address these imbalances for ethical [3] and economic reasons [19]. Availability and affordability are both poor in India [20,21]. Dependent on pharmaceutical companies and government [3], they may be improved by more equitable funding. Recent policies to improve healthcare in India have focused on public health infrastructure, universal healthcare coverage, increased health workers in rural settings and reducing out-of-pocket expenditure [16−22]. In relation to access to drugs, the requirements to ‘Improve quality, performance, eﬃciency, and accountability of public and private health systems’ and ‘Have in place mechanisms to check and control the use of perverse incentives by pharmaceutical and biotechnology companies for health-care providers’ have been recognized as priorities [21−22]. Policies to broaden access to medicines have included the Government of India initiative, ‘Jan Aushadhi’, a countrywide chain of medical stores to make generic and other drugs available at reasonable prices, by bulk purchasing and drug price control, but acceptability and feasibility of these proposals remains unknown.

The issues that face Indian healthcare are complex and multiple actions would be required to make access more equitable [22]. Service delivery, adequate finance, improvements in health information systems and good governance are all considered crucial to healthcare improvement in India, alongside the need to improve drug access [22]. Improved access to medicines requires broadening of access to generic drugs, as well as lowering the cost and improving the supply of patented drugs. The Rashtriya Swasthya Bima Yojna (RSBY) scheme was established by the Government of India in 2008 and aims to provide health insurance cards worth 30,000 rupees (~US$ 500) per year to all citizens living below the poverty line. National policies such as RSBY and universal healthcare coverage have indirectly aimed to reduce out-of-pocket health expenditure which may improve access to medicines, but no specific national strategy exists to directly improve access to medicines.

Alternative funding for pharmaceutical innovation may offer sustainable and equitable access to novel medicines [17], in the context of declining research and development and stagnant sales [23,24]. The Health Impact Fund (HIF), originally described by philosopher Thomas Pogge and health economist Aidan Hollis in 2008, is an academic policy proposal, stimulated by failure of current reward mechanisms to address access to medicines [25,26]. As a global agency underwritten by governments, the HIF would offer pharmaceutical innovators the option to register any new product. Registration would entitle the innovator to receive, for a defined period (e.g. 10 years) a share of fixed remuneration from a reward pool. Working within TRIPS [17,27], the HIF would enable pharmaceutical companies to be paid based on the measured global health impact of their drugs [17] (Appendix 1).

The HIF is still in development via an international, interdisciplinary team (including AB as a Medical Advisor and DP as a Scientific Board member). It requires testing of acceptability and feasibility as well as modelling of potential implications before any policy implementation. Global health impact would be predicted based on preliminary trial data (‘efficacy’) and reward would be based on actual impact (‘effectiveness’). The exact nature of global health impact assessment is yet to be finalized, but would require: (i) modelling and extrapolation of trial efficacy data to a particular population at country or regional level; (ii) estimation of ‘real world’ effectiveness using actual outcome data; and (iii) calculation of ‘reward’ based on the impact of the drug (Appendix 2). All firms would have the option to register a new drug with the HIF and, by doing so, must sell the drug at the lowest possible cost, which would be determined by the lowest feasible cost of production and distribution and acceptability of all stakeholders, including governments, payers, patients and pharmaceutical companies [17]. The financial reward would be a portion of pooled resources, financed by governments and other donors, during the drug’s patent [17,28]. The HIF would, theoretically, drive development of drugs that have use in diseases with a high burden worldwide. This may improve access to novel medicines within India, by reducing costs and improving supply. A sum of $6 billion per year (provided by partner countries supporting the HIF) has been suggested as the minimum amount necessary to allow the HIF to support development of two drugs per annum, sustaining a stock of 20 medicines [27].

Previous surveys and studies have considered access to medicine [29] but the HIF and other proposals to improve access to medicines often lack evidence before implementation, with limited evaluation of proposed benefits. The acceptability of the HIF (which is a policy proposal in development) to key stakeholders is unknown and will affect its feasibility in India and other countries. We therefore conducted the first stakeholder analysis of the HIF as a means for improving access to medicines in India with the following aims:

1. To determine key stakeholder views regarding access to medicines in India.
2. To evaluate acceptability of the HIF.
3. To assess potential barriers and facilitators to the HIF as a policy.

The HIF is intended as a global policy, funded by governments, but this study was designed to specifically assess Indian perspectives in relation to it.

## Methods

### Design and setting

This study used stakeholder analysis as its primary methodology, defined as ‘a process of systematically gathering and analysing qualitative information to determine whose interests should be taken into account when developing and/or implementing a policy or program’ [30]. Due to time and resource constraints, but also due to the geographic concentration of major stakeholders in Delhi, recruitment was in this setting in India. In consultation with Research and Information Systems for Developing Countries (RIS, which is based within the Ministry of External Affairs, Government of India, and has specific expertise in trade and intellectual property-related matters, including in relation to healthcare) we identified key actors. We divided stakeholders into six categories: pharmaceutical sector, public health, intellectual property law, government, non-governmental organizations (NGOs) and research funding organizations. We explored influence and power by questions focusing on the alliances and resources of the stakeholders [31–33]. Based on inductive interpretation of the interview, stakeholders’ power/influence was categorized for their sector, according to whether they had high or low influence on policy relating to access to medicines (e.g. the HIF), ‘specifically the degree to which they were able to place the issue on the public or political agenda; influence legislation; actively participate in major decision-making fora; and mobilize on this issue’ as per a previous stakeholder analysis [32]. We ensured diversity of stakeholder perspectives in the research setting (New Delhi, India) in order to represent a range of sectors and a spectrum of opinion with respect to the HIF [34]. Ethical approval was obtained from the Independent Ethics Committee at the Centre for Chronic Disease Control, India, and the Internal Research Ethics Committee, University of Birmingham, UK.

### Sample

We supplemented purposive recruitment of participants with snowball sampling [34] and used the iterative nature of theoretical sampling [35,36]. Instead, we focused on diversity of recruitment. We initiated recruitment through networks from RIS in Delhi, emailing the Participant Information Sheet and HIF information (Appendix 1), and then snowballed. The information sheet included reasons for this research project and conflicts of interest. Of 49 potential participants contacted, 23 responded (47%). Fourteen face-to-face interviews were conducted in English between 19 February and 5 March 2015 (nine individuals declined due to unwillingness or lack of availability) (Appendix 3). Therefore, overall participation rate was 14/49 (29%).

### Data collection

Informed consent was gained from each participant (Appendix 4). No relationship was established with participants prior to study initiation. The interview guide (Appendix 5) was based on literature regarding health policy in India, the HIF and on topics derived from the study aims. It was not pilot tested but agreed by all authors. We conducted semi-structured face-to-face interviews using this stakeholder analysis framework [30], aiming to recruit from each of the six stakeholder groups. Interviews were conducted at the office of the stakeholder or a location convenient for them which would facilitate audio recording. We asked for stakeholder views regarding: access to medicine in India, interest and opinions with respect to the HIF, suggestions for improving access to medicines, as well as potential alliances and resources to translate the HIF into reality. The interviews lasted 20–60 minutes and were all, except one, audio-recorded using a digital tape recorder. Notes were made during the interviews. The interviews were conducted by PM, as a medical student, following basic training in qualitative research at the University of Birmingham and mentorship from senior researchers involved in qualitative research (AB, DP and RS).

### Analysis

Following transcription of the recorded interviews, we assessed transcripts for any typological errors and removed any confidential information. Transcripts were not returned to participants for comment or correction. We used directed content analysis [37] with principal themes of the HIF and access to medicines. In particular, with respect to access, we considered availability (the supply and demand), affordability (the drug price and patients’ ability to pay), accessibility (physical access) and adaptability (the relationship between patients perceptions and what is being offered) as per previous studies of access to medicines [4]. Initially, text relevant to each theme was highlighted. The highlighted passages of text were then coded using pre-determined codes in NVivo 10.0. This process continued until all highlighted data were fully coded. We then conducted a more inductive sub-coding [38], whereby all coded data were sub-coded for further detail. Coding was undertaken by PM and AB.

A reflexive approach was ensured by completion of a diary throughout the process, and the primary researcher (PM) could question interpretations to ensure no conflict of interest and bias minimization. Quotations used as evidence in the results have been edited (in terms of syntax) for flow [39]. On the basis of power/influence and interest (classified by the coder) in the HIF in line with previous studies [32], each participant was classified as a ‘defender’ (high interest, low power), ‘bystander’ (low interest, low power), ‘driver’ (high interest, high power) or ‘blocker’ (low interest, high power) with respect to the HIF as a policy (Figure 1). This terminology was developed by AB and PM. A defender would have high motivation to defend the HIF from criticism but low influence. A bystander is low on influence and interest and unlikely to promote or criticize the HIF. A driver is a powerful supporter of the HIF. A blocker is an influential opponent to the HIF. The assigned category was not checked with the participant. The study was designed and reported to meet consolidated criteria for reporting qualitative research (COREQ) [40] and the relevant sections of the COREQ checklist are described in the relevant sections above.

## Results

Most stakeholders felt there were significant issues with access to medicines in India, yet their views regarding the proposed HIF were mixed and, although positive in general, there was scepticism with multiple perceived disadvantages. Few stakeholders demonstrated total opposition to the HIF and when opposition was noted, it was focused on amendment of the HIF rather than the aim of improving access to medicines per se.

### Views on access to medicines in India

#### Access to most citizens is poor and heterogeneous despite a strong domestic pharmaceutical industry

The prevailing idea that access is poor for the majority of Indians was common. Although participants from pharmaceutical and government sectors agreed, they focused on different aspects, for example:

[The pharmaceutical] industry has done remarkably well to meet the country’s need and at prices which are ... I mean you may say compared to Indian income may not be affordable still but if you compare with the world prices these are very, very low prices. (P1; Pharmaceutical; Driver)

The view that the ‘[pharmaceutical] industry has done remarkably well’ is to be expected from a representative of that industry, yet a candid view on the issue with access was still given. The price may be cheap, but even that relatively low price is still, for many, out of reach when the nation ‘has a vast amount of the population which is in the category of absolutely poor or extremely poor … so whatever price drugs are available … they won’t be able to purchase these medicines’ (P6; Government; Bystander). Government and ministerial participants corroborated the opinion of the pharmaceutical industry that despite there being issues with affordability, as the public health expert states, the ‘situation is improving significantly with the state governments taking an active role in expanding free supply of medicines’ (P10; Government; Driver). This goes to the heart of access to medicines in India; that healthcare is a state-based issue and there is large inter-state heterogeneity:

Contrast is the name that can be given in many health situations or for that matter any matter of health or any walk of life in India. So in some states there may be reasonably strong access but in some states it may be just not that great. (P9; Public Health; Defender)

Access to medicines includes medicines that are off-patent, and produced by generic companies, as well as those on-patent. Many of the participants referred to overall access being poor but a few highlighted the issue of highly expensive patented medicines.

For example, ‘when it comes to medicine and particularly the patented medicines … it is basically coming from abroad. Most of it is coming from abroad and they have put the prices as very high’ (P11; Pharmaceutical; Defender).

The mention of the high cost for patented medicines must be put into the context of the low purchasing power of many Indians. If these *‘*extremely poor’ citizens are unable to purchase any medicines, as participant 6 suggested, then the novel patented medicines are well beyond their purchasing capability. In other words, the scale of the price is less important than the need for free supply of medicine in India.

#### Branded generics inflate prices

The issue with affordability is not solely with expensive patented medicines but several participants also identified an issue with ‘branded generics’ (versions of a drug that are bioequivalent to the original product, but are marketed under another company’s brand name):

You have had what is known as branded generics … this is the main challenge I would feel. This is perpetuated because the prescriptions are largely written in brand name and not in generic name ... If prescription is in a brand name then the patient almost mistakes a brand name for a medicine. (P10; Government; Driver)

This gives rise to a situation where ‘access may be stratified on information flow*’* (P12; Research funder). If the patient is unable to challenge the doctor, who prescribed branded and not generic drugs, due to lack of awareness, then unnecessary higher drug costs ensue, and ‘though we have a lot of generics in India, still they are expensive*’* (P2; Public health; Defender).

#### Out-of-pocket expenditure dominates

Out-of-pocket expenditure and catastrophic health expenditure were perceived as major issues:

Drugs are meant to be free in public healthcare but are often unavailable and so patients buy out-of-pocket from local shops themselves. (P9; Public Health; Defender)

Lot of our reports … very clearly indicate that one chronic ailment, one acute case, the person is indebted. He has to sell land and even we have seen family members becoming destitute. (P6; Government; Bystander)

### Views on the health impact fund

Table 1, the stakeholder analysis matrix, represents stakeholder opinions with respect to the HIF’s policy implementation. The majority (12/14; 86%) of the participants were unaware of the HIF prior to being invited to participate in the research. The stakeholder analysis showed that one participant (P14; NGO) was a potential ‘blocker’ of the HIF, with four participants classified as ‘drivers’ (P1; Pharmaceutical; P7; Public health; P10; Government; P13; NGO). Two participants were considered ‘bystanders’ (P5; NGO; P6; Government) and the remaining seven interviewees were ‘defenders’. Other than two individuals, all other participants highlighted affordability as a major determinant of drugs in India. Availability was the second most commonly cited issue (in 9/14). No participants listed affordability as the only issue in access to medicines.

**Table 1.** Stakeholder analysis matrix comparing the interest of each actor in the Health Impact Fund with their perceived power and influence on health policy setting.

PARTICIPANT NUMBER	SECTOR	POWER/INFLUENCE	INTEREST	CATEGORY	DOMAIN OF ACCESS TO MEDICINES			
					availability	affordability	accessibility	adaptability
1	Pharmaceutical	High	High	Driver	x	x		
2	Public Health	Low	High	Defender	x	x	x	
3	IP law	Low	High	Defender	x	x		x
4	Public Health	Low	High	Defender		x	x	
5	NGO	Low	Low	Bystander	x		x	x
6	Government	Low	Low	Bystander	x	x		
7	Public Health	High	High	Driver	x	x		x
8	Research Funding	Low	High	Defender		x		x
9	Public Health	Low	High	Defender	x	x		
10	Government	Low	Low	Driver		x		x
11	Pharmaceutical	Low	High	Defender		x	x	x
12	Research Funding	Low	High	Defender	x	x		
13	NGO	High	High	Driver	x		x	
14	NGO	High	Low	Blocker		x		x

#### Perceived positives of the health impact fund

The underlying aim of the HIF (to increase access to medicines) was generally praised, for example:

For a practising public health professional in India, if you get some superior quality of drug at the lowest price, and assuring its availability, by and large, in a reasonably big way…and there is some regulatory mechanism that which drugs are to be there and which drugs not: that is a dream situation. (P9; Public health; Defender)

But do you think it is feasible? (Interviewer)

But whether it is feasible or not, that is a different issue. (P9; Public health; Defender).

Support for the HIF’s ideals, but reservations about its capabilities, were common to many participants.

The frequently held view was that India should, and would, be able to commit to financing such an initiative; for example: *‘*I am sure the developed countries will put in money and India should not be found lagging behind much’ (P10; Government; Driver). This builds on a sense of pride by the Indian nation; to not be *‘*lagging behind’ but to contribute to global initiatives that aim to improve access to medicine. This view was corroborated by other participants: ‘there are India, South Africa, Brazil, the BRICS countries… So because they are the emerging donors also … they may be interested in some of these global activities’ (P9; Public health; Defender). The view that funding should be possible is encouraging.

#### Perceived negatives of the health impact fund policy concept

The strongest criticism of the HIF was that it may not have value in the poorest settings ‘in countries such as sub-Saharan Africa, for example, where the public health challenges are huge. In fact, you don’t require new and innovative drugs, you are just required to improve the health systems over there, use the best available drugs which can make a big change. What would be the role of Health Impact Fund?’ (P4; Public health; Defender).

A major concern was that the HIF was too small to have an impact: ‘Even this $6 billion for the HIF, with this looking at diseases with market, to me it does not make sense because this is too modest a figure’ (P5; NGO; Bystander).

Moreover, there was scepticism about how impact assessment may actually function:

I don’t think the Health Impact Fund is very well fleshed out. How do you sort of define these steps for reward and impact measurement and who pays for it and how does it all go in? (P14; NGO; Blocker)

This Health Impact Fund, they need to do some more work not just with government but have a dialogue with the industry. (P1-Pharmaceutical; Driver)

Perhaps the most interesting critique of the HIF was that, by working within current worldwide intellectual property frameworks, i.e. TRIPS, it was less likely to improve access to medicines:

*I am not sure whether this can be a good model because the current patent system is broken… Unless we do something about breaking that monopoly we are not going to see medicines made available.* (P2; Public health; Defender).

*‘* *…..the strong intellectual property regime… would get legitimacy… This has been one of the questions which the Health Impact Fund has always faced, and other proposals have faced.’* (P6; Government; Bystander).

The HIF would only offer pharmaceutical companies an alternative; they could still continue monopoly marketing, which was perceived to be against equitable global access to medicines:

*‘* *I am not very optimistic of a state instrumentality really solving this problem of the greed of pharma companies to make money.’* (P10; Government; Driver)

Indian pharmaceutical representatives argued that a research base must exist in India, in order for funding post-marketing (as outlined in the HIF) to be tenable:

‘*We don’t have experience of developing new original molecules. Therefore, the developing country industry does not have access to these funds’* (P1; Pharmaceutical; Driver).

This would create a situation where the HIF may be *‘skewed towards big pharma because it seems to be supportive of the big pharma’s argument in terms of we innovate and we produce these drugs and so we should get the profits’* (P4; Public health; Defender).

Several participants argued that patented medicines were not the priority:

*‘I am not saying that patented medicines don’t have an important role but I am saying that look… problems will exist but you can largely overcome the problems in Indian context by settling the generic medicine pricing and use’* (P10; Government; Driver).

### Suggestions to alter the health impact fund to best improve access to medicines

#### Focus on diseases with no market/ neglected diseases

The limited size of the estimated yearly funding pool, $6 billion, and resulting need for more focus were frequently emphasised:

‘*Prioritise the areas you want to tackle because there are so many different countries, diversities, demographies and differing needs… as the name suggests “Health Impact Fund”, to me you can create impact if you are focused on a few well-chosen priorities’* (P13; NGO; Driver).

‘*If I am the CEO of the Health Impact Fund… I would look at those diseases which could make an impact where industry is unable to do, where there is a market failure… the entire area of neglected diseases requires attention… companies will require to be incentivised to do these trials… Those are areas which I would like to focus because this is not a big amount. It is only 6 billion’* (P5; NGO; Bystander).

#### Fund innovation directly

Indian pharmaceutical companies may lack sufficient research and development capabilities to create new molecules. Amendment of the HIF to offer partial funding of research and development was suggested:

‘You must fund research scientists who are working in the lab, as they say from the bench to the bedside, you cannot just focus on the bedside or in between, you have got to focus some [money] on the bench as well. So part of the fund, I think, should be… to kick-start enterprise and research*’* (P8; Ministry and government sector).

For me the Health Impact Fund is not something that companies will go for. They will probably try and resell something that they already have, to the Health Impact Fund … so offering the money at the end of the chain really doesn’t help, it is the work that you do at the beginning of the chain that really makes a difference …. Like for example a bio-marker for a TB test that will help you get TB in urine or blood is not something that the companies will do. (P14; International healthcare NGO)

## Discussion

This is the first stakeholder analysis regarding the HIF and access to medicines in India. We note three key findings. First, access to medicines is perceived to be a major problem in India by all stakeholders, involving branded and generic drugs and it is not only the problem of pricing, which is the main target of the HIF. Second, although several stakeholders supported the HIF as a vehicle to improve access to medicines, there were major concerns about its scaleability, feasibility and impact, mainly because drug pricing alone does not equate to drug access. Third, in order to overcome barriers to the HIF, novel drug-related health policies may need to be more radical, e.g. working outside the existing IP regime, targeting generic as well as branded drugs, or extending to research and development.

A poorly funded public health system drives many Indians to buy branded drugs out-of-pocket and previous research [41] has emphasized heterogeneity in access [42]. However, it is of great interest that members of all stakeholder groups, including the pharmaceutical sector, feel strongly that the status quo is problematic. In the Global Burden of Disease Study, India ranked 154th among 195 countries for healthcare access and quality [43]; therefore, the problem is wider than access to medicines. Moreover, there is wide regional variation across India in disease burden and disease outcomes [44]. Although there is likely to be support for policies tackling access to medicines, it is unlikely that a single health policy reform (such as the HIF) can tackle all or even most of the problems around access to medicines [45]. Therefore, as the WHO highlights, priority-setting is required [41], and branded medicines may not be the highest priority. In India, generic drugs are a major component of the pharmaceutical market and with the advent of multiple ‘fixed-dose combination’ preparations (or ‘polypills’), there is scope for generic or non-patented medications to be included in rewards which are dependent on impact assessment (such as the HIF).

There is stakeholder support for change, but our analysis (Table 1) suggests a wide range of perceived power and interest of stakeholders, even within stakeholder group (e.g. government or NGO) regarding the HIF. Although the majority were in favour rather than against, it is clear that policies which improve access to medicines, whether to gain support of government or the pharmaceutical sector, will require consideration of more than drug affordability. Any policy aiming to tackle access to medicines should be multi-sectoral and involve consultation of multiple, different stakeholders, in order to maximize support and likelihood of success, as well as using available information regarding affordability, availability and other components of access [4]. Reassuringly, several stakeholders felt that the HIF may not be radical enough, suggesting again that policy reform has to be cross-sectoral and cannot be a ‘stand-alone’ measure. The IP regime was repeatedly highlighted as a barrier to improving access, as was the lack of available funding for research into new molecular entities in India. There are other policy solutions which have been ‘field-tested’ in India and in other countries, including local pharmaceutical production [46], managed entry agreements [47] and generic drug prioritization [48]. Pharmaceutical policy has been shown to vary by country income, and no policy is likely to be a ‘one-size-fits-all’ [49]. Before the HIF can be implemented, policy details such as the exact nature of health impact assessment and reward mechanisms need to be finalized. Moreover, research and modelling of the pharmaceutical market (branded versus generic, pre- and post-trial drugs, common versus rare diseases, well-funded versus poorly funded diseases) are essential to maximize feasibility and ensure credibility [4].

There are several limitations inherent in our study. This was a relatively small qualitative study and only a limited number of stakeholders from each sector were interviewed. The absence of a representative from the Ministry of Finance was a specific limitation that meant data on the feasibility of funding for healthcare initiatives was poor. The stakeholders that participated in the study may have been more interested in the policy, thereby agreeing to an interview, than those who did not reply to the recruiting emails. The participants were all in senior positions and may be somewhat distanced from the ‘reality on the ground’, but, conversely, these individuals were more likely to have a ‘big picture’ policy perspective. Perhaps most importantly, the interviewer was a medical student from the UK, who was not experienced in qualitative research, especially in the Indian context, despite appropriate training and mentorship arrangements in the UK and India. Conversely, compared with having an Indian expert or an experienced researcher in access to medicines conducting the research, the bias in interviews was likely to have been minimised.

The stakeholders may have not given detailed analysis on the HIF due to unfamiliarity with its proposals and a potential unwillingness to disregard a potential policy that they have not fully researched, which may introduce bias. However, the framework did enable a detailed appreciation of both each stakeholder’s initial views and interest in the policy as well as assessing their power, giving important information to then move the policy forward. We solely focused on the HIF, but the findings may be relevant to development of other policies regarding access to medicines in India with probable relevance but not necessarily generalizability to other countries. The stakeholders and particular barriers and facilitators for access to medicines are country-specific and therefore further studies are needed in other countries. Although two authors (AB and DP) have advisory roles in relation to the HIF, a balanced view was possible and reporting of the mixed views of stakeholders, including concerns about significant limitations show that the risks of bias and conflict of interest were low.

The HIF is one novel solution to the lack of access and innovation in medicines that requires pilot data and field testing. Several international initiatives over the last two decades, including the Gavi (the global vaccine alliance), the Global Fund for TB, Malaria and HIV/AIDS, numerous public-private partnerships and research and development funding schemes have aimed to improve access to medicines for neglected tropical diseases. Compared with these solutions, which have tended to be disease-focused, short-term and vertical in nature, the HIF may offer a system-wide incentive to improve drug innovation based on the greatest global health impact, but it has its own problems of focusing on drugs rather than health systems, and patented rather than generic drugs.

## Conclusion

Access to medicines is perceived to be an important issue in India, requiring urgent policy reform, according to our stakeholder analysis. The Health Impact Fund, a proposal to improve access to medicines by incentivising pharmaceutical companies to produce drugs with the greatest global health impact, is one such suggestion, but may not be far-reaching enough to be successful in its aim of improving access to medicines. The importance of context-specific quantitative and qualitative data (via stakeholder analysis) prior to policy reform and implementation is highlighted by our study.

## Paper context

*Current knowledge:* Access to medicines in India requires innovative policies. The Health Impact Fund (HIF) is a potential solution whereby pharmaceutical companies would be paid based on measured health impact of drugs.

*Findings:* The HIF and other new policies should, at least initially, focus on diseases with limited or no market, and incorporate direct investment in research.

*Implications:* Policies to improve access to medicines can be more radical, e.g. targeting generic and branded drugs, or extending to research and development.

## Appendix 2.

**Health impact assessment in the HIF (quoted and adapted from Banerjee, A., Hollis, A and Pogge, T. The Health Impact Fund: incentives for improving access to medicines. Lancet. 2010;375(9709):166–169)**

The health impact assessment will draw on the same types of information that agencies such as the National Institute for Health and Clinical Excellence (NICE) now use to make recommendations about listing new drugs, but will aim to update this information with data for use of the drug and information from new practical trials. No method of health impact assessment could be perfect and indisputable in its measurements or the estimates produced from it. However, although these drawbacks are serious, the real comparison is not with a perfect system but with the present situation. In the UK, where drug insurance is universal and NICE assesses the cost-effectiveness of medicines, the present situation can be viewed as a reward system in which each sale of a drug creates a reward equal to the difference between the price and the cost of manufacture and distribution. The size of the reward per pill is fixed on the basis of information provided to NICE at the time of drug approval, and is typically not modified to show new information. Thus, the UK system is essentially a simple reward system in which the reward equals the price–cost margin multiplied by the number of units sold.

The HIF’s health impact assessment would improve on this standard in at least five important ways:

- physician survey data about actual use of medicines
- evidence for drug use in medical practice through practical trials
- patient usage data through surveys of patients who were prescribed the drug
- outcomes data
- data from post-approval phase 4 clinical trials, especially comparative trials.

## Appendix

**Appendix 3.** Participants

Participant number	Organisation	Sector	Position	Date of interview
P1	Indian Pharmaceutical Alliance	Pharmaceutical	Senior	19/02/2015
P2	Public Health Foundation of India	Public Health	Senior Public Health Specialist and Assistant Professor	19/02/2015
P3	SKS Law Associates (patent law)	IP law	Senior	21/02/2015
P4	Centre for Chronic Disease Control	Public Health	Senior	24/02/2015
P5	Open Source Drug Discovery	NGO	Senior	24/02/2015
P6	Institute for Studies in Industrial Development	Government	Senior	25/2/2015
P7	World Health Organization	Public Health	Senior	26/02/2015
P8	Biotechnology Industry Research Assistance Council (BIRAC)	Research Funding Organization	Senior	01/03/2015
P9	Indian Institute of Public Health	Public Health	Senior	02/03/2015
P10	National Pharmaceutical Pricing Authority	Government	Senior	02/03/2015
P11	Indian Drug Manufacturers Association	Pharmaceutical	Senior	03/03/2015
P12	Wellcome Trust	Research Funding Organization	Senior	04/03/2015
P13	NATHEALTH-Healthcare Foundation of India	NGO	Senior	05/03/2015
P14	Medecin Sans Frontieres	NGO	Senior	05/03/2015
